# A Rare Case of Squamous Cell Carcinoma of the Gallbladder Presenting as a Bleeding Colonic Mass

**DOI:** 10.7759/cureus.114942

**Published:** 2026-08-21

**Authors:** Oziegbe Egbo, Ojevwe H Egbo, Favour A Onyeka, Faith I Omoregie

**Affiliations:** 1 Internal Medicine, Benson Idahosa University, Benin City, NGA; 2 Anatomical Pathology, Benson Idahosa University, Benin City, NGA; 3 Internal Medicine, Ripple Medical Center, Benin City, NGA

**Keywords:** colonoscopy, gallbladder carcinoma, grade 4 hemorrhoids, histology, immunohistochemistry

## Abstract

Gallbladder carcinoma (GBC) is the most common malignancy of the biliary tract, with adenocarcinoma being the predominant histological subtype. Squamous cell carcinoma (SCC) is rare, accounting for approximately 1%-4% of gallbladder carcinomas, and is characterized by aggressive local invasion and poor prognosis. We report a 53-year-old man with a one-year history of recurrent painless lower gastrointestinal bleeding and a six-month history of progressive constipation, abdominal bloating, and unintentional weight loss. His bleeding had previously been attributed to a known grade 4 hemorrhoid. Colonoscopy revealed a friable, bleeding mass at the hepatic flexure in addition to non-bleeding grade 4 hemorrhoids. Histopathological examination of the colonic biopsy demonstrated moderately differentiated SCC. Immunohistochemistry was positive for cytokeratin 7 (CK7) and p63, and negative for cytokeratin 20 (CK20), hepatocyte paraffin 1 (HepPar-1), and alpha-fetoprotein (AFP). Contrast-enhanced computed tomography (CT) subsequently demonstrated a large heterogeneous gallbladder mass with direct invasion of the adjacent liver and hepatic flexure, rendering the tumor unresectable. The integrated clinicoradiological and pathological findings supported a diagnosis of locally advanced gallbladder SCC with direct extension into the colon. The patient received gemcitabine and oxaliplatin chemotherapy, followed by endoscopic retrograde cholangiopancreatography (ERCP) with biliary stent placement and palliative surgical management for persistent lower gastrointestinal bleeding. This case highlights the diagnostic challenge posed by gallbladder SCC presenting as a bleeding colonic mass. Squamous carcinoma identified in a colonic lesion should prompt consideration of an extra-colonic primary, with immunohistochemistry interpreted alongside clinical and radiological findings. Persistent gastrointestinal bleeding warrants thorough evaluation even when an apparent anorectal source is present.

## Introduction

Gallbladder carcinoma (GBC) is an uncommon but highly lethal malignancy of the biliary tract and is the most common primary malignancy of the biliary system, accounting for approximately 80%-95% of biliary tract cancers [1,2]. Its incidence varies markedly across geographical regions, with the highest rates reported in South-Central Asia, Eastern Asia, and South America [3]. In contrast, GBC is relatively uncommon in sub-Saharan Africa, with studies from Nigeria reporting a low incidence compared with other regions [4,5]. The disease frequently presents at an advanced stage because early GBC is often clinically silent or associated with nonspecific symptoms, contributing to its poor prognosis [6].

The development of GBC is associated with chronic inflammation and persistent mucosal injury. Established risk factors include gallstones, porcelain gallbladder, chronic cholecystitis, and chronic biliary infections such as *Salmonella* species [1,2]. Histologically, adenocarcinoma accounts for more than 90% of GBCs, whereas squamous cell carcinoma (SCC) is a distinctly uncommon histological subtype, accounting for approximately 1%-4% of malignant gallbladder tumors [7]. Gallbladder SCC has been reported to exhibit more aggressive clinical features, including larger tumor size, more extensive local invasion, and poorer survival compared with adenocarcinoma [7]. Its rarity and aggressive behavior make recognition of this histological variant particularly important.

GBC commonly spreads through direct invasion of adjacent organs, lymphatic and vascular routes, and peritoneal dissemination, with the liver, regional and non-regional lymph nodes, and peritoneum among the commonly involved sites [8]. Involvement of the colon is distinctly uncommon, with only isolated reports describing colonic involvement by GBC [8,9]. Reported cases demonstrate that colonic involvement may present with gastrointestinal bleeding and may clinically and endoscopically resemble a primary colorectal malignancy [8,9]. In addition to metastatic disease, direct extension from a locally advanced gallbladder tumor may result in involvement of the adjacent hepatic flexure. Such an atypical pattern of presentation may obscure the primary site and complicate diagnostic evaluation.

The identification of SCC in a colonic biopsy presents an additional diagnostic challenge because primary colorectal SCC is itself extremely rare. Histological identification of SCC in the colon does not, by itself, establish a primary colorectal origin; secondary involvement from another primary tumor must first be excluded [10]. The differential diagnosis therefore includes primary colorectal SCC, metastatic SCC from an extra-colonic primary site, and direct extension from an adjacent squamous malignancy. Establishing the site of origin requires integration of histomorphological findings with immunohistochemistry, cross-sectional imaging, and the overall clinical context [11]. This distinction is particularly important because the primary site determines disease staging, treatment strategy, prognosis, and multidisciplinary management.

Lower gastrointestinal bleeding is frequently attributed to common anorectal conditions, particularly hemorrhoidal disease, and this may influence initial diagnostic reasoning [12]. Although hemorrhoids are a common cause of rectal bleeding, persistent or recurrent bleeding should prompt appropriate reassessment, particularly when accompanied by other concerning features such as altered bowel habit, weight loss, anemia, or constitutional symptoms. Inadequate evaluation of rectal bleeding remains a recognized clinical problem, with a substantial proportion of patients not undergoing complete endoscopic or radiological assessment following initial presentation [11]. In patients with established hemorrhoids, attributing persistent bleeding solely to hemorrhoidal disease may therefore delay recognition of an underlying gastrointestinal malignancy.

We report a rare case of previously undiagnosed pure SCC of the gallbladder presenting as a bleeding hepatic-flexure colonic mass in a patient with coexisting grade 4 hemorrhoids. The case illustrates the diagnostic difficulty of distinguishing an unusual gallbladder primary tumor with colonic involvement from a primary colorectal malignancy and highlights the importance of correlating histopathological and immunohistochemical findings with cross-sectional imaging. It also underscores the need for careful reassessment of persistent gastrointestinal bleeding and a multidisciplinary approach when an apparently benign explanation does not adequately account for the clinical picture.

## Case presentation

A 53-year-old male engineer was referred to our center with a one-year history of recurrent, painless rectal bleeding and a six-month history of progressive constipation, severe abdominal bloating, and unintentional weight loss. He had previously received care at multiple facilities, where his bleeding had been attributed to a known grade 4 hemorrhoid. There was no history of jaundice, dark urine, pale stools, fever, or abdominal pain.

On physical examination, the patient appeared cachectic and markedly pale, with tachycardia. The abdominal examination was unremarkable, with no palpable masses or organomegaly. A digital rectal examination confirmed the presence of a grade 4 hemorrhoid with no active bleeding. Cardiovascular, respiratory, and central nervous system examinations were essentially normal.

Laboratory evaluation (Table 1) revealed severe anemia. Other hematologic parameters were within normal limits. Serologic testing for human immunodeficiency virus (HIV), hepatitis B virus (HBV), and hepatitis C virus (HCV) was negative. Liver function tests, including alkaline phosphatase (ALP), aspartate aminotransferase (AST), and alanine aminotransferase (ALT), were within normal ranges. Serum electrolyte analysis demonstrated hyponatremia, while urea and creatinine levels were normal. Coagulation studies showed a mildly prolonged prothrombin time. Serum tumor markers, including carbohydrate antigen 19-9 (CA 19-9) and carcinoembryonic antigen (CEA), were within normal limits.

**Table 1:** Key laboratory, endoscopic, and imaging findings ALP: alkaline phosphatase, AST: aspartate aminotransferase, ALT: alanine aminotransferase, CA 19-9: carbohydrate antigen 19-9, CEA: carcinoembryonic antigen, RBG: random blood glucose, HBsAg: hepatitis B surface antigen, HCV: hepatitis C virus, HIV: human immunodeficiency virus, INR: international normalized ratio, IHC: immunohistochemistry, CK7: cytokeratin 7, CT: computed tomography, GI: gastrointestinal, USS: ultrasound sonography

Investigations	Result	Reference range	Interpretation
Pre-chemotherapy			
Hemoglobin	6.5 g/dL	11.0-17.0 g/dL	Low
ALP	185 U/L	80-306 U/L	Normal
AST	67 U/L	10-46 U/L	Elevated
ALT	35 U/L	7-49 U/L	Normal
Total bilirubin	0.6 mg/dL	0.2-1.2 mg/dL	Normal
Direct bilirubin	0.2 mg/dL	0.1-0.4 mg/dL	Normal
Total protein	6.3 g/dL	6-8 g/dL	Normal
Serum albumin	3.9 mg/dL	3.5-5.2 mg/dL	Normal
CA 19-9	0.01 U/mL	≤40 U/mL	Normal
CEA	3.40 ng/mL	0-10 ng/mL	Normal
RBG	98 mg/dL	70-140 mg/dL	Normal
HBsAg	Negative	-	Normal
HCV	Negative	-	Normal
HIV	Negative	-	Normal
Prothrombin time	17 seconds	10-15 seconds	Elevated
INR	1.4	0.8-1.5	Normal
Serum sodium	129 mmol/L	135-155 mmol/L	Low
Serum potassium	3.8 mmol/L	3.6-5.5 mmol/L	Normal
Serum bicarbonate	23 mmol/L	23-29 mmol/L	Normal
Serum chloride	99 mmol/L	95-108 mmol/L	Normal
Serum creatinine	1.0 mg/dL	0.9-1.3 mg/dL	Normal
Serum urea	31 mg/dL	10-55 mg/dL	Normal
Colonoscopy (macroscopic)	Multinodulated colonic mass at the hepatic flexure	-	Abnormal
Colonic biopsy (histology)	Moderately differentiated squamous cell carcinoma	-	Diagnostic
Hepatic biopsy (histology)	Moderately differentiated squamous cell carcinoma	-	Diagnostic
Colonic biopsy (IHC)	Positive for CK7 and p63	-	Diagnostic
Hepatic biopsy (IHC)	Positive for CK7 and p63	-	Diagnostic
Abdominal CT scan	A large heterogenous mass arising from the gallbladder fossa with direct invasion into the adjacent right hepatic lobe and the hepatic flexure of the colon	-	Diagnostic
During chemotherapy			
ALP	67 U/L	80-306 U/L	Normal
AST	136 U/L	10-46 U/L	Elevated
ALT	147 U/L	7-49 U/L	Elevated
Total bilirubin	2.4 mg/dL	0.2-1.2 mg/dL	Elevated
Direct bilirubin	0.7 mg/dL	0.1-0.4 mg/dL	Elevated
Total protein	6.6 g/dL	6-8 g/dL	Normal
Serum albumin	3.9 mg/dL	3.5-5.2 mg/dL	Normal

**Figure 1:**
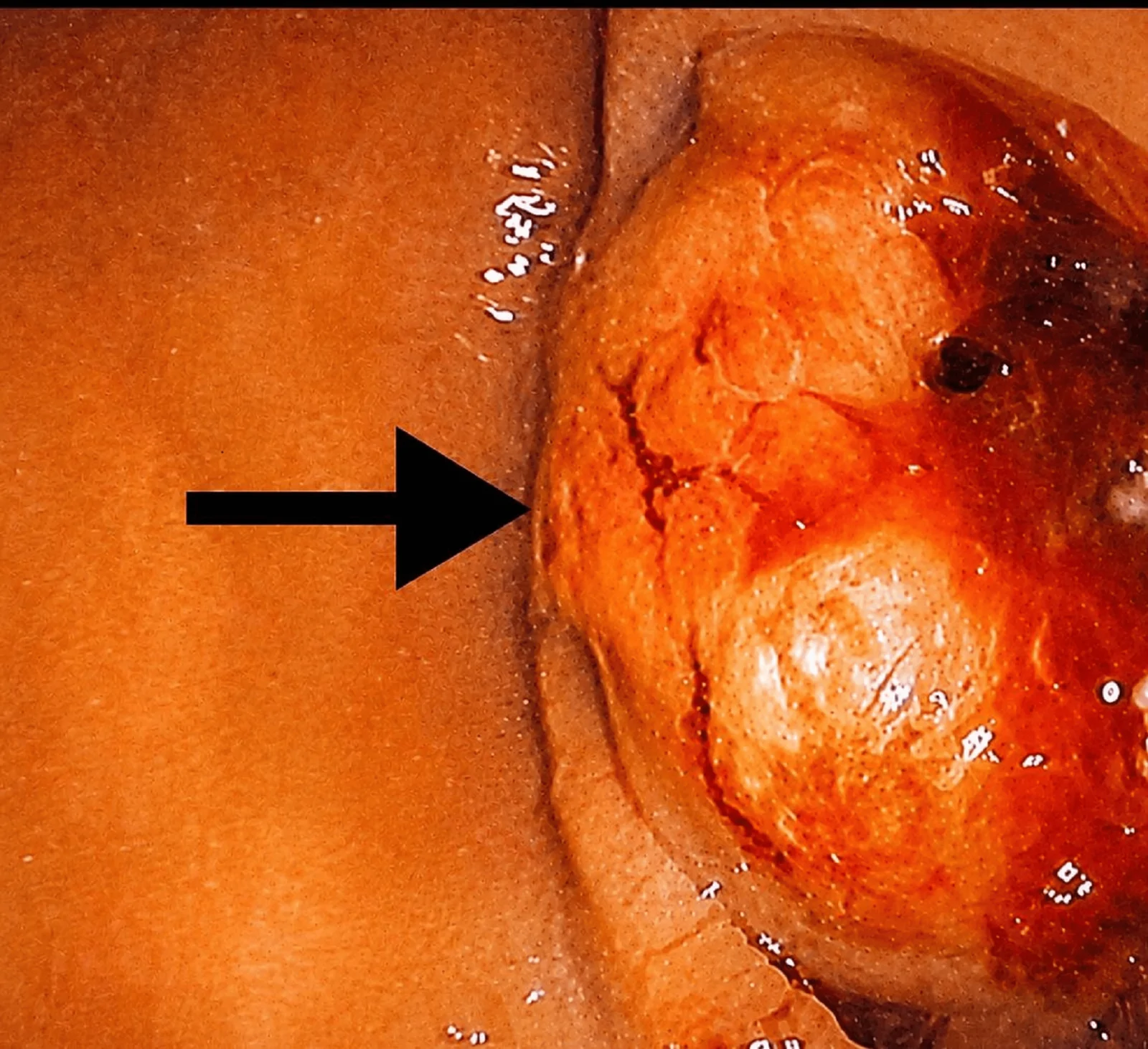
Colonoscopic view of the hepatic flexure An exophytic colonic mass is noted partially occluding the colonic lumen. The black arrow highlights the friable tumor with active bleeding from its surface.

A colonoscopy identified a multinodulated colonic mass at the hepatic flexure, which was friable and bled on contact, in addition to the previously noted grade 4 hemorrhoid. Multiple biopsies of the mass were obtained (Figure 1).

**Figure 2:**
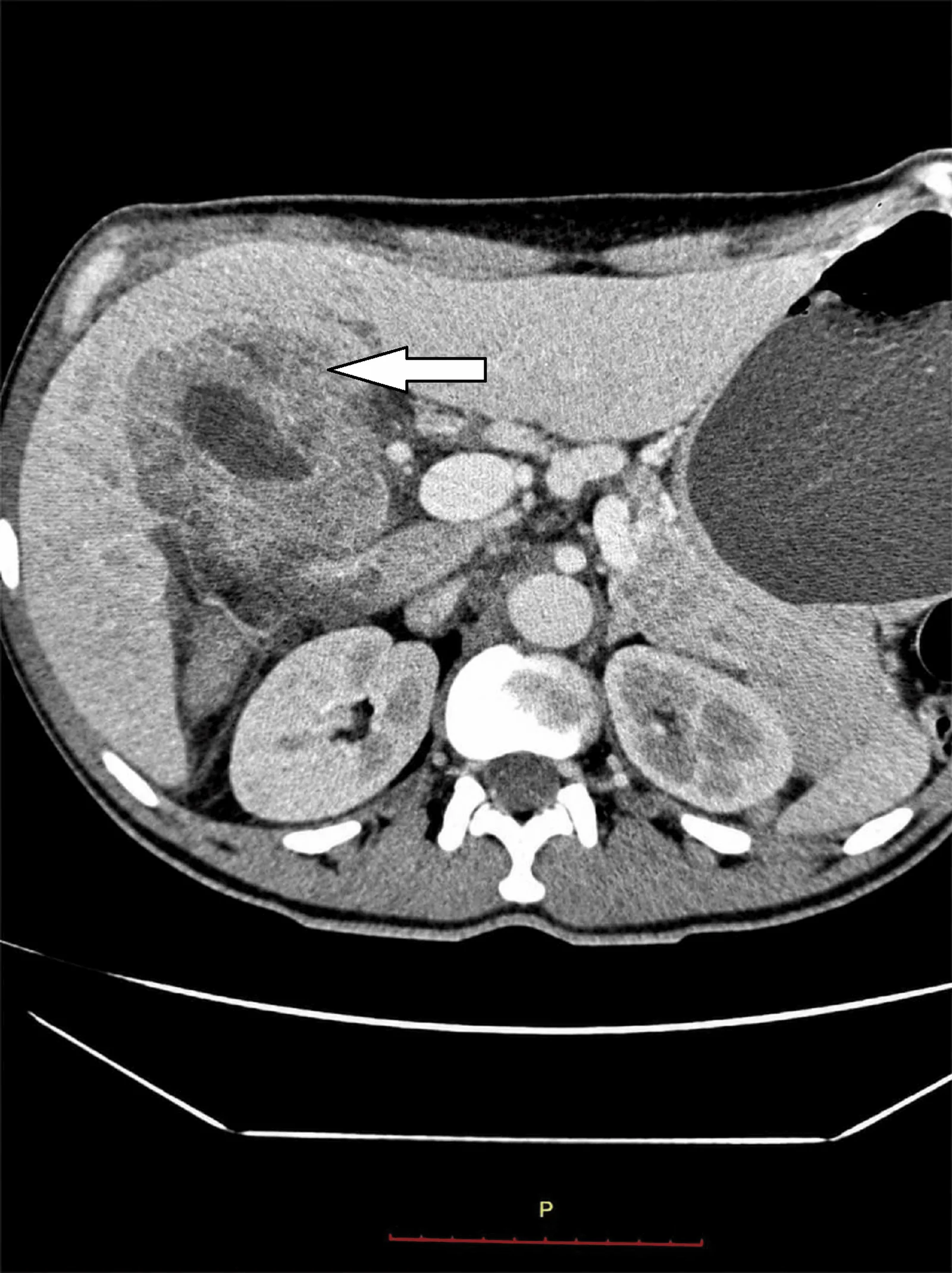
Axial contrast-enhanced computed tomography scan of the abdomen The arrow highlights a large, heterogeneous, contrast-enhancing mass originating from the gallbladder fossa with direct contiguous extension into both the hepatic parenchyma and the adjacent colon.

Contrast-enhanced computed tomography (CT) of the abdomen revealed a large heterogeneous mass arising from the gallbladder, measuring 10.16 cm × 9.51 cm × 6.54 cm, with contrast enhancement. The mass demonstrated direct invasion into the adjacent right hepatic lobe and the hepatic flexure of the colon. No gallstones were seen. Based on imaging findings, the tumor was deemed unresectable (Figure 2).

**Figure 3:**
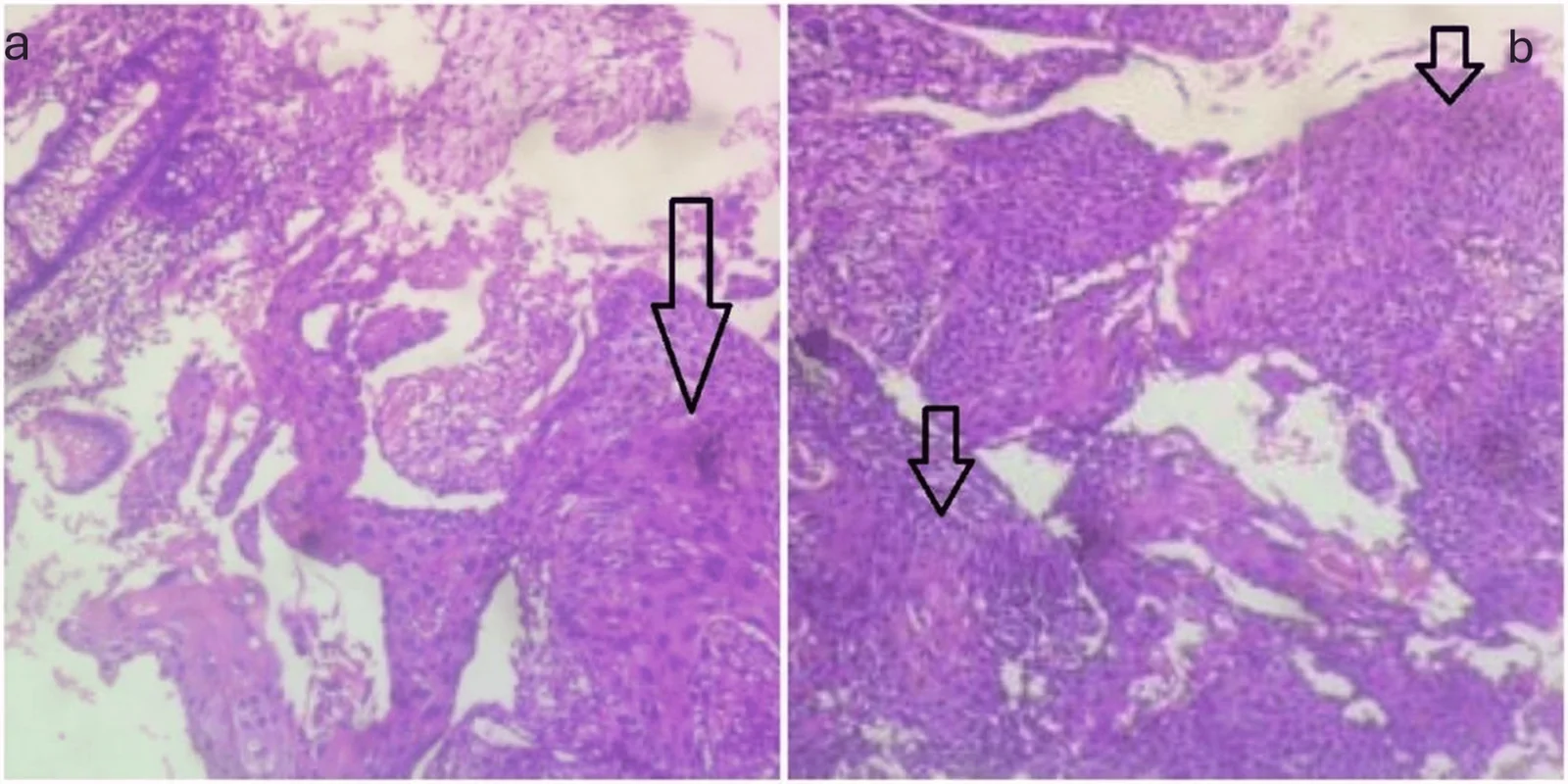
Histopathological sections of the colonic mass tissue (a and b) H&E-stained sections demonstrating a malignant epithelial neoplastic lesion composed of dysplastic squamous cells arranged in small sheets, nests, cords, anastomosing trabeculae, and papillae. The black arrows highlight direct neoplastic infiltration into the lamina propria and muscularis mucosae, accompanied by significant gland resorption and peritumoral inflammation. Frequent abnormal mitotic figures, alongside patchy areas of hemorrhage and necrosis, are also present. H&E: hematoxylin and eosin

Histopathological examination of both the colonic and ultrasound-guided hepatic biopsy specimens demonstrated moderately differentiated squamous cell carcinoma (Figure 3).

**Figure 4:**
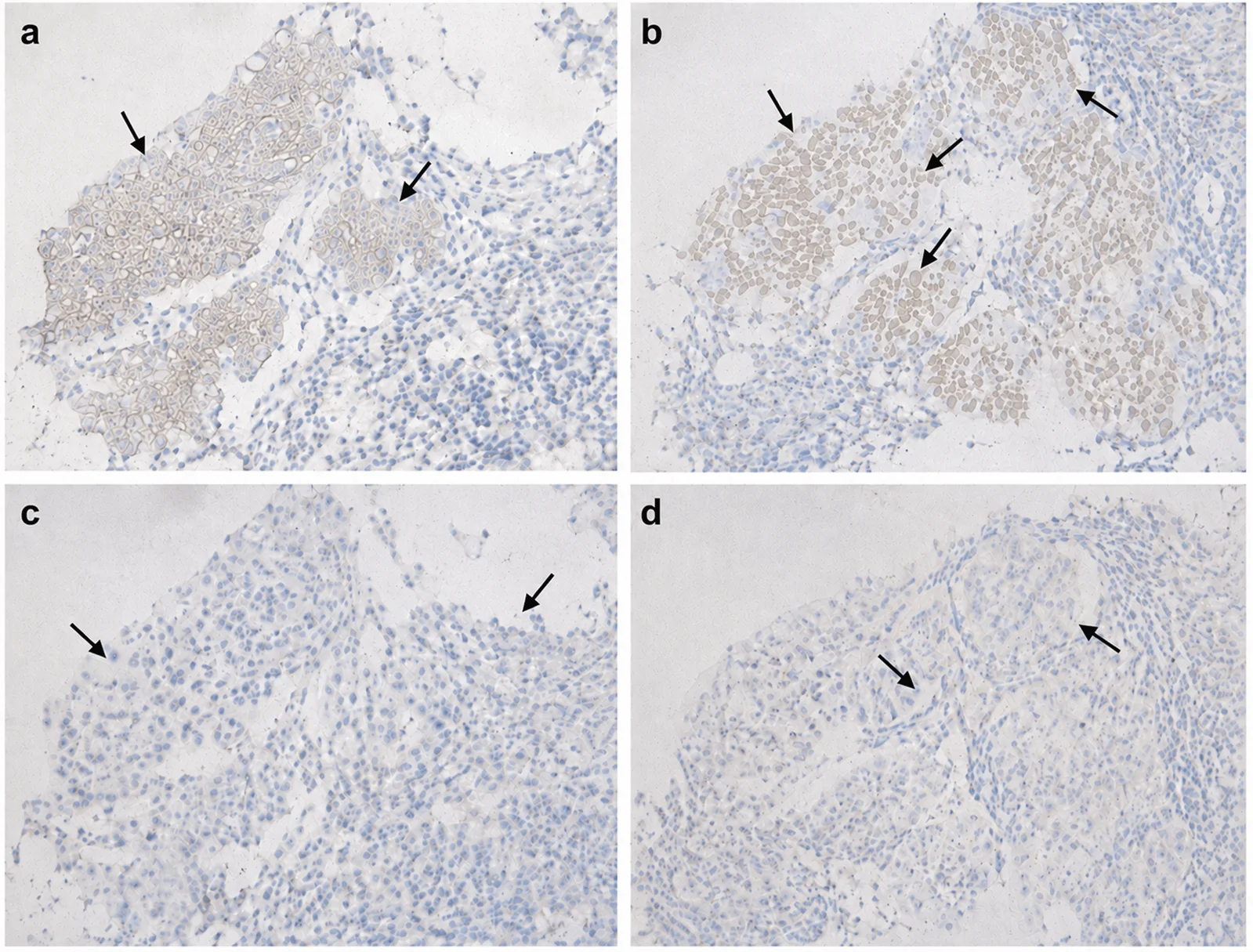
Immunohistochemistry staining profile of the tumor cells (a) CK7 immunostaining demonstrates strong positive cytoplasmic and membranous brown chromogenic staining in the tumor cells (arrows). (b) p63 immunostaining demonstrates strong nuclear brown chromogenic staining in the tumor cells (arrows). (c) CK20 immunostaining is negative, with absence of brown chromogenic staining in the tumor cells (arrows). (d) HepPar-1 immunostaining is negative, with absence of brown chromogenic staining in the tumor cells (arrows). Original magnification: ×400 CK7: cytokeratin 7, p63: tumor protein 63, CK20: cytokeratin 20, HepPar-1: hepatocyte paraffin 1

Immunohistochemical analysis revealed positivity for cytokeratin 7 (CK7) and tumor protein p63, and negativity for cytokeratin 20 (CK20) and hepatocyte paraffin 1 (HepPar-1) (Figure 4).

The patient was commenced on combination chemotherapy with gemcitabine and oxaliplatin. Following the third cycle, repeat liver function tests were deranged. In view of the rising bilirubin levels, endoscopic retrograde cholangiopancreatography (ERCP) with biliary stent placement was performed. Post-procedure, the patient developed persistent lower gastrointestinal bleeding, requiring recurrent blood transfusions. Owing to ongoing hemorrhage and clinical deterioration, the patient subsequently underwent an exploratory laparotomy, during which an end-to-end ileotransverse anastomosis was performed, along with an excision of the hepatic flexure tumor.

## Discussion

Gallbladder carcinoma (GBC) is the most common malignancy of the biliary tract but remains relatively uncommon worldwide, accounting for less than 2% of all cancers [13,14]. Adenocarcinoma comprises approximately 90%-95% of cases, whereas pure squamous cell carcinoma (SCC) represents only 1%-4%, making it one of the rarest histological subtypes of gallbladder malignancy [7]. Despite its rarity, SCC demonstrates a distinctly more aggressive biological behavior than conventional adenocarcinoma, characterized by rapid tumor growth, extensive local invasion, larger tumor size at diagnosis, and poorer overall survival [7,13]. A recent systematic review confirmed that patients with gallbladder SCC typically present with more advanced T-stage disease and have significantly worse outcomes than those with adenocarcinoma, largely because of extensive invasion into adjacent organs before diagnosis [7,14].

Although the precise pathogenesis of gallbladder SCC remains uncertain, several mechanisms have been proposed. Chronic inflammation secondary to cholelithiasis, recurrent cholecystitis, or chronic *Salmonella* carriage is thought to induce squamous metaplasia of the gallbladder epithelium, which may subsequently undergo malignant transformation [13,15]. Alternatively, squamous differentiation may arise from pre-existing adenocarcinoma through metaplastic change, explaining the more frequent occurrence of adenosquamous carcinoma compared with pure SCC. These mechanisms may partly explain the aggressive proliferative capacity of squamous tumors, which tend to expand locally rather than metastasize early through lymphatic dissemination. This propensity for contiguous extension often results in invasion of the liver, duodenum, stomach, or colon before distant metastatic spread becomes clinically apparent [7,13,15].

The clinical presentation of gallbladder SCC is generally indistinguishable from conventional GBC and commonly includes right upper quadrant pain, anorexia, weight loss, jaundice, or a palpable abdominal mass. Gastrointestinal bleeding is exceptionally uncommon. Our patient presented with recurrent painless lower gastrointestinal bleeding for almost one year and was repeatedly managed for grade 4 hemorrhoids before the underlying malignancy was identified. This presentation represents an important diagnostic pitfall because the presence of an obvious anorectal source may delay further investigation of proximal colonic pathology. Bleeding from malignant gastrointestinal tumors generally results from tumor ulceration, friability, and neovascularization, all of which were evident during colonoscopy in this patient [7].

Colonic involvement by gallbladder carcinoma is itself uncommon and may occur either through direct invasion of the hepatic flexure or, much less frequently, true metastatic implantation. Published reports describing colonic involvement have predominantly involved conventional adenocarcinoma, with presentations including bowel obstruction, perforation, or incidental radiological findings [7,13]. Reports involving pure SCC remain exceedingly rare. Compared with previously published cases, our patient differed in both the duration and mode of presentation, manifesting recurrent lower gastrointestinal bleeding without classic hepatobiliary symptoms despite harboring a large unresectable tumor [16]. This prolonged diagnostic interval illustrates how atypical presentations may obscure the underlying diagnosis, particularly in resource-limited settings where common anorectal disorders are far more prevalent than hepatobiliary malignancies [7,13,16].

One of the major diagnostic challenges in this case was determining the primary site of the squamous carcinoma identified on colonic biopsy. Primary squamous cell carcinoma of the colon is itself an exceptionally rare tumor and remains a diagnosis of exclusion after eliminating metastatic SCC from other organs and direct extension from adjacent structures. Histologically, our tumor demonstrated CK7 and p63 positivity, with CK20, HepPar-1, and alpha-fetoprotein (AFP) negativity, findings that supported biliary epithelial differentiation while excluding hepatocellular carcinoma and making conventional colorectal adenocarcinoma unlikely. Nevertheless, immunohistochemistry alone cannot establish the site of origin. Markers such as CK7 and p63 demonstrate epithelial lineage and squamous differentiation rather than organ specificity. Likewise, CK20 negativity does not exclude primary colorectal SCC because this tumor frequently lacks conventional colorectal immunophenotypes. Consequently, clinicopathological correlation with high-quality cross-sectional imaging was essential in establishing the gallbladder as the primary tumor [13,15,17].

An additional diagnostic consideration is distinguishing direct invasion from true metastatic colonic disease. Radiologically, our patient’s large gallbladder mass demonstrated continuous extension into the adjacent hepatic flexure without evidence of a separate discontinuous colonic lesion, favoring locally advanced contiguous invasion rather than isolated intraluminal metastasis. Histopathology from the colonic biopsy confirmed squamous carcinoma but could not independently differentiate between these mechanisms. This distinction has important implications because direct invasion generally reflects locally advanced T4 disease, whereas discontinuous colonic metastasis indicates systemic dissemination. Our case therefore emphasizes the importance of multidisciplinary review involving radiologists, pathologists, and hepatobiliary surgeons when evaluating unusual colonic squamous lesions [7,13].

Management of gallbladder SCC remains challenging because prospective treatment guidelines are lacking. Complete R0 surgical resection remains the only potentially curative treatment; however, most patients present with unresectable disease because of extensive local invasion. Systemic chemotherapy using gemcitabine-based regimens is frequently employed, although evidence specific to SCC is limited and outcomes remain poor. In our patient, unresectability at presentation necessitated palliative chemotherapy followed by ERCP for biliary decompression and subsequent palliative surgery for persistent hemorrhage. This clinical course highlights both the aggressive natural history of gallbladder SCC and the importance of early recognition before extensive locoregional extension precludes curative surgery [7,15].

In summary, this case expands the limited literature on gallbladder SCC involving the colon by illustrating an exceptionally unusual presentation with prolonged recurrent lower gastrointestinal bleeding. It also highlights several important diagnostic lessons. First, squamous carcinoma identified on colonic biopsy should prompt a careful search for an extra-colonic primary tumor. Second, immunohistochemistry should always be interpreted in conjunction with radiological findings because no single immunomarker reliably establishes gallbladder origin. Finally, persistent gastrointestinal bleeding despite an apparent anorectal source warrants complete colonic evaluation, particularly when accompanied by constitutional symptoms such as weight loss and progressive anemia. Early multidisciplinary assessment may facilitate earlier diagnosis and improve opportunities for potentially curative intervention.

Table 2 compares published cases of gallbladder SCC involving the colon or adjacent colorectal structures.

**Table 2:** Comparison of published cases of gallbladder squamous carcinoma involving the colon or adjacent colorectal structures SCC: squamous cell carcinoma, GI: gastrointestinal, IHC: immunohistochemistry, CT: computed tomography, ERCP: endoscopic retrograde cholangiopancreatography

Study	Histology	Colonic involvement	Initial presentation	Diagnostic approach	Management	Key point
Alpuerto et al. (2017) [18]	Pure SCC	Direct invasion of adjacent gastrointestinal organs (duodenum/stomach); locally aggressive disease	Abdominal pain, weight loss	CT + histopathology + IHC	Surgical exploration	SCC demonstrates marked contiguous local invasion.
Jin et al. (2019) [13]	Pure SCC	Extensive local invasion into the liver and abdominal cavity	Abdominal pain	Imaging + pathology	Palliative management	Large tumor burden and poor prognosis despite diagnosis.
Idris et al. (2020) [16]	Pure SCC	Colon invaded late during disease progression	Biliary obstruction	Imaging + pathology	Chemoradiotherapy + supportive care	Colon involvement reflects advanced disease and poor survival.
Present case	Pure SCC	Direct invasion of hepatic flexure presenting as bleeding colonic mass	One-year recurrent painless lower GI bleeding initially attributed to hemorrhoids	Colonoscopy, IHC, CT, and multidisciplinary review	Gemcitabine/oxaliplatin, ERCP, palliative surgery	Unique presentation mimicking primary colorectal bleeding and illustrating the importance of clinicopathological correlation.

## Conclusions

This case demonstrates an exceptionally rare presentation of primary SCC of the gallbladder masquerading as a bleeding colonic mass in a patient with coexisting grade 4 hemorrhoids. It adds to the limited literature on colonic invasion by GBC and describes the diagnostic challenges posed by atypical presentations. Despite the poor prognosis associated with gallbladder SCC, heightened clinical suspicion, thorough evaluation of persistent gastrointestinal bleeding, and early multidisciplinary collaboration are essential to facilitate timely diagnosis and optimize patient outcomes.
